# Spatial Variation of *b*-Values and Their Relationship with the Fault Blocks in the Western Part of the Tibetan Plateau and Its Surrounding Areas

**DOI:** 10.3390/e22091016

**Published:** 2020-09-11

**Authors:** Hamid Hussain, Zhang Shuangxi, Muhammad Usman, Muhammad Abid

**Affiliations:** 1 Department of Geophysics, School of Geodesy and Geomatics, Wuhan University, Wuhan 430079, China; shxzhang@sgg.whu.edu.cn; 2 Key Laboratory of Geospace Environment and Geodesy of Ministry of Education, Wuhan University, Wuhan 430079, China; 3 Collaborative Innovation Center of Geospace Information Science, Wuhan University, Wuhan 430079, China; 4 Physical Science and Engineering Division, King Abdullah University of Science and Technology, Thowal 23955, Saudi Arabia; muhammad.usman@kaust.edu.sa; 5 School of Ocean and Earth Science, Tongji University, Shanghai 200092, China; abid_geoscientist@tongji.edu.cn

**Keywords:** Tibetan Plateau, *b*-value, strike-slip faults, earthquakes, Mc (magnitude of completeness), Depth histogram

## Abstract

The Tibetan Plateau is considered to be one of the best natural laboratories for seismological research. This study sought to determine the spatial variations of *b*-values in the western part of the Tibetan Plateau, along with its surrounding areas, and the relation with the region’s fault blocks. The study region lies within 27–36.5° N, 78–89° E, and its fracture structure consists of strike-slip faults, as well as normal and thrust faults. A catalog record from 2009–2019 provided 4431 well-centered earthquakes that varied in magnitude from 0.1 to 8.2 M. The record was obtained from China’s seismological network, which is capable of recording low magnitudes to analyze *b*-values in the study area. The key findings of this study are as follows: (1) the range of earthquake depth in the region was 0–256 km, with the depth histogram showing a high frequency occurrence of shallow earthquakes in the area; (2) a time histogram showed that the major earthquakes occurred between 2014–2015, including the notable 2015 Gorkha earthquake (M = 8.2); (3) the *b*-value computed in the study area was 0.5 to 1.6, but in most of the study area, the *b*-value ranged from 0.6 to 0.9, which was a low to intermediate value, due to the presence of strike-slip faults in the central part of the study area and underthrusting in the region (south of the study area); and (4) a high *b*-value was found in the northwestern and eastern regions of the area, which proved that the area is prone to small earthquakes in the near future. The study also showed that the central and southern areas of the study region had low to intermediate *b*-values, meaning that it is prone to destructive and massive earthquakes with high magnitudes, such as the Gorkha earthquake (southern part of the study area). Low *b*-values revealed the degree of variation in rock properties, including large stress and strain, a fractured medium, a high deformation rate, and large faults. Small *b*-values were observed when the stress level was high in the investigated region, which might be used to predict a massive high-magnitude earthquake in the near future.

## 1. Introduction

China has suffered from earthquakes for centuries, such as the 1556 Shaanxi earthquake (8.0 magnitude) [1], the 1976 Tangshan earthquake (7.8 magnitude) [2], and the 2008 Wenchuan earthquake (7.9 magnitude) [3]. Each of these earthquakes resulted in a massive loss of human life and property. In China, the Tibetan Plateau is the region with the most intense neotectonic movement and seismic activity. The Tibetan Plateau has a devastating record of earthquakes with widespread human casualties and socioeconomic damages, mainly due to its high-level earthquake activity and severe tectonic deformation. The 1950 Assam—Tibet earthquake (8.6 magnitude) engulfed many human lives and is the largest recorded earthquake caused by continental collision rather than subduction [4]. The 1952 Damxung earthquake struck Tibet with a magnitude of 7.5. The focal mechanism of the 8 November 1997 Manyi earthquake in northern China indicated that the earthquake occurred on a left-lateral strike-slip fault [4].

**Figure 1:**
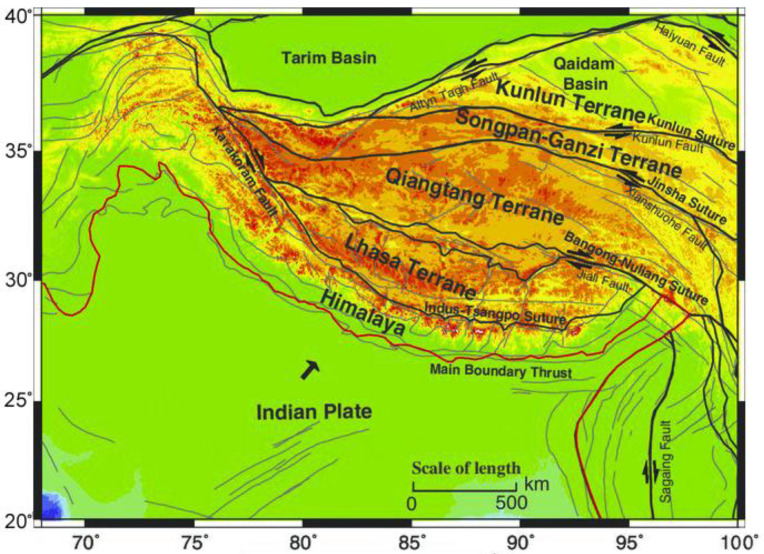
Tectonic map of the Tibet—Qinghai Plateau. The area is divided into different terrains [5].

The youngest orogenic belt in the Tibetan Plateau resulted from a collision between the Indian and Eurasian Plates, which was followed by the closure and subduction of the Neo-Tethys Ocean. The Tibetan Plateau’s average elevation exceeds 5000 m and might be the largest and highest place on the Earth’s surface. To the north, it is surrounded by deserts in the Qaidam Basin and Tarim Basin, and the Himalayan and Karakoram basins lie to its south and west, respectively (Figure 1).

The Tibetan Plateau is divided into three zones: Northern, Central, and Southern Tibet [5]. This division is due to the shortening in the north, shearing in the center, and underthrusting in the south. Figure 1 shows that from north to south, there are five terrains: The Qilian terrain, the Kunlun—Qaidam terrains, the Songpan—Ganzi—Hoh Xil terrain, the Qiantang terrain, and the Lhasa terrain [5].

These terrains are separated by several suture zones [6]. Most of the shock resulting from the collision of the Indian Plate with the Eurasian Plate was absorbed by the Himalayas. The Tibetan Plateau is an important region to better understand the theory of plate tectonics and orogenic evolution on Earth, as it is composed of several thin and fine continental pieces within the eastern Tethyan domain [7]. The Tibetan Plateau is seismically active and has a thick crust, while the lithosphere is relatively thin. Thus, the crust of the plate is approximately twice the thickness of normal continental crust. On the Tibetan Plateau from block to block, however, the crustal thickness varies [8].

**Figure 2:**
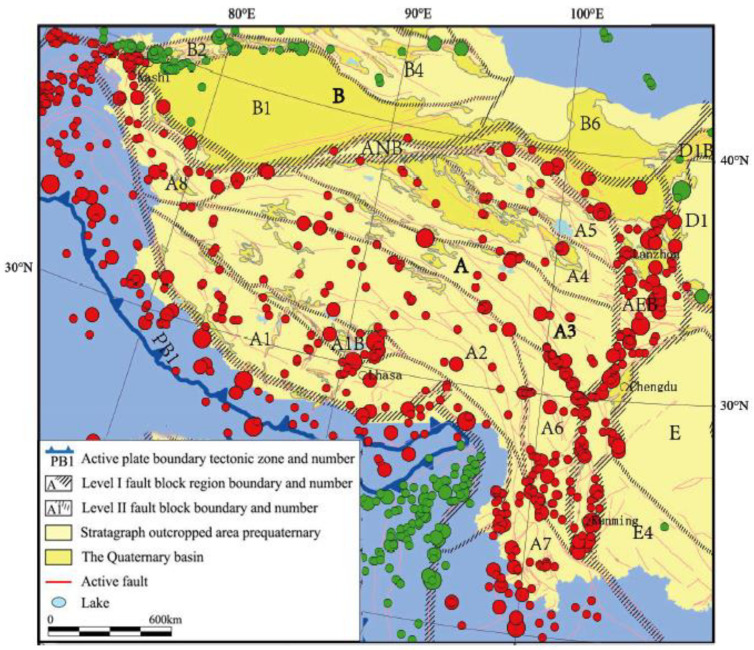
Map showing the fault zones along the margins of the blocks. The circles show earthquakes with different magnitudes ranging in size from M6.0 to M8.9, modified after Deng et al. 2014 [10]. Blocks labeled with an A constitute the Tibetan Plateau structural region or fault-block region; ANB is the north boundary zone of A, and AEB is the east boundary zone of A.

Beneath the Himalayan and Lhasa blocks, the crustal thickness is about 60 km. The thickness of the crust is about 75 km beneath the Qiangtang block [9]. The Tibetan Plateau has a complex geometry and deformation style. Therefore, it is challenging to study the area fault by fault, as it is a huge area where much deformation has occurred. The plateau is divided into different blocks, as shown in Figure 2. These blocks are separated by different fault zones—i.e., the Honghe—Jiali—Pangongtso, Xianashuihe—Yushu—Margaicaka, East Kunlun, and West Qinling—Northern Qaidam fault zones—of which some of the faults are simple to understand, while others are difficult to understand. The following fault-bounded block regions are shown and labeled on Figure 2: (A1) Lhasa, (A2) Qiangtang, (A3) Bayan-har, (A4) East Kunlun—Qaidam, and (A5) the Qilian Mountains [10].

According to Deng et al. [10], most earthquakes with large magnitudes occur within Tibetan Plateau boundaries or at the boundaries of active tectonic zones in intraplate fault blocks. There is a correlation between the plate boundary zone and intraplate earthquake series. According to kinematics, not only is compression from the Indian Plate occurring toward the northeast, but each block in the Plateau’s fault block region tends to move southeastward at different slip rates [11]. The slip rates are different throughout the plateau. For example, the Jiali Fault has a slip rate of 10–15 mm yr^−1^, whereas the Xianshuihe Fault has a slip rate of 13–15 mm yr^−1^ [12]. Towards the north of the Yunnan—Sichuan fault block, the southeastward slip rate decreases. In each block region, there are differences in certain types of tectonics, which are characterized by different motion characteristics. These characteristics reveal the complexity of the plateau’s crustal structure, with a multilayered structure of alternate soft and hard rock layers. The catalog from China’s Earthquake Network Center (CENC) relies on the arrival time of motion within the regional seismic network, which provides a good constraint on focal depths for earthquakes within the study area. However, the geophysical results collected are not consistent because of scarce station coverage and complex velocity structure, to which the understanding of the Tibetan Plateau’s evolution, especially western Tibet, is still incomplete. However, compared to western Tibet, much research has been done in eastern Tibet.

## 2. Background of the *b*-Value

An earthquake is considered one of the most terrible natural disasters that threatens human survival and development. It is challenging to determine the time, location, and magnitude range for future seismicity. Many phenomena in earthquake predictions (i.e., seismic wave velocity, gravity, resistivity, electricity, and magnetic fields) have been studied as event precursors. However, it is difficult to evaluate if a general method for earthquake forecasting is statistically successful because the time, location, and size of an earthquake must be specified [13]. Sometimes, there is a resolution problem due to a relatively large number of events, which are otherwise mostly rare or of limited number in the area. Many studies have been carried out on the temporal and spatial variations of *b*-values as a predecessor for upcoming large events. The *b*-value was discovered to be associated with rock heterogeneity, thermal gradients, and width depth. The *b*-value has an inverse relationship with stress, as was found with low *b*-value anomalies at locations of asperities and high *b*-value anomalies in Alaskan and New Zealand subduction zones. Moreover, *b*-values decrease with depth in continental crust [14,15,16,17,18,19]. Schorlemmer et al. [20] reported high *b*-values in areas with normal faults, intermediate *b*-values in strike-slip regions, and low *b*-values associated with thrust events [20]. The regional average estimate of the *b*-value usually equals one (*b*~1.0), and many factors can interfere with average *b*-values [21,22]. For areas with greater geological complexity, a high *b*-value was reported, indicating multiple fracture areas. Therefore, a low *b*-value indicates nonuniformities of a cracked medium and a low degree of variation in rock properties, such as large stress and strain, a fractured medium, a high deformation rate, and large faults.

## 3. Previous Studies of *b*-Value in Tibet

According to Jiu et al. [22], the *b*-value encountered in Xinjiang was 0.773. In the study area, the *b*-values decreased as depth increased. At 5–10 km, the *b*-value was 0.810. At a depth of 26–33 km, the *b*-value was 0.763. According to Chandrani et al. [23], the *b*-value in southern Tibet ranged from 0.6 to 1.2. Chandrani et al. [23] also concluded that the *b*-value decreased with depth. According to Liu et al. [24], in the Longmenshan area, the *b*-value showed a significant boundary above 15 km where *b*-values were higher. Below this depth, the *b*-value decreased. The spatial variation of the *b*-value showed an increase (i.e., reduced stress) in the Wenchuan rupture zone [25]. Many studies have considered the spatial and temporal variations of *b*-values in seismic zones, but most of the work has been done on specific locations and does not relate findings to the structural geology of the area. In this study, we calculated the spatial variations of the *b*-value in the western part of the Tibetan Plateau and its surrounding areas extending from north to south, as well as their relationship with the fault blocks in the study area. The large spatial extent of the region and the relatively small regional dataset would not afford us the ability to resolve the temporal variation of the *b*-value.

Based on previous studies, we deduced that the *b*-value has great significance and substantial practical value. Therefore, the *b*-value was used as a structural index in the study area and reflected the distribution characteristics of earthquake magnitude frequency. Further, the *b*-value has been widely used in earthquake prediction. Knowing the recurrence (or cycle) period of earthquakes in an area is an essential parameter for evaluating seismic risk. Therefore, we explored and measured the recurrence period of earthquakes in the Tibetan Plateau from the interrelated aspects of seismology, geology, and deformation measurement.

## 4. Earthquake Data

**Figure 3:**
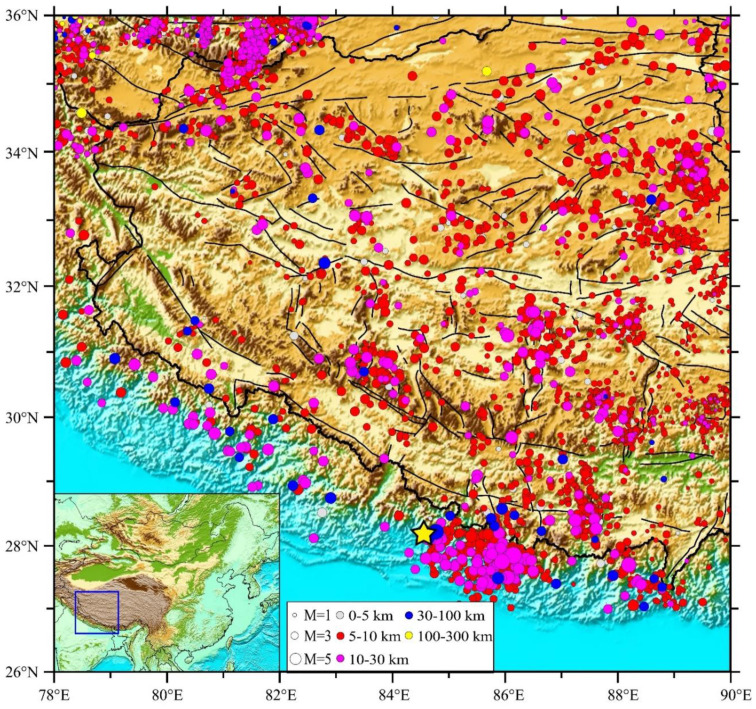
Map showing the study area. The size and color of the circles show earthquakes with different magnitudes and different depths, respectively. The star shows the Gorkha earthquake with magnitude 8.2, which occurred in 2015.

We collected the data to study the *b*-value in the Tibetan Plateau and its surrounding areas from the China Seismological Center, which included small magnitudes measured from 2009 to 2019. The data were limited because there were few seismic stations in the area; the Plateau is vast and is unreachable for geological and geophysical studies because of a lack of road infrastructure and inclement weather. The epicenters of earthquakes used in this study lied within the area bounded by 27–36.5° N and 78–89° E, at the western part of the Tibetan Plateau (Figure 3).

In this study, we investigated 4431 earthquake events (Table 1). The range of depth of the earthquakes used in this study ranged from 0–256 km. The study area included four large earthquake events with magnitudes greater than 7 that occurred between 2014 and 2015. The earthquake events analyzed had a minimum magnitude of 0.1 and a maximum magnitude of 8.2. The only earthquake with a magnitude greater than 8 (shown in Table 1) in the study area was the Gorkha earthquake (28.15° N, 84.65° E), which happened on 25 April 2015 and had a focal depth of 20 km (recorded by the China Earthquake Networks Center). According to the U.S. Geological Survey, the magnitude of the Gorkha earthquake was 7.8 [24].

## 5. Analysis Method

To study the earthquake size distribution in the Tibetan Plateau, we followed the Gutenberg-Richter power law [9] (Log_10_N = *a* − *b*M), which is one of the most useful relations in seismology, where 10*^a^* is the total number of earthquakes, *b* is the relative earthquake size distribution, and N is the number of earthquakes with a magnitude equal to or greater than M [26]. This relation has been verified for global and regional seismicity in different global seismic zones. According to the Gutenberg-Richter formula, a lower *b*-value means that larger earthquakes will dominate over smaller earthquakes. A higher *b*-value means that there is dominance of small earthquakes over large earthquakes. The *b*-value reflects the relationship between large and small earthquakes, as well as the stress state of different parts of the medium [27]. We propose that *b*-values can be used as precursors to large earthquakes.

This study was carried out to examine the *b*-value in space and its relationship with fault blocks in the region. The first step for determining the spatial variation of the *b*-value was to calculate the magnitude of completeness (Mc). In this study, we checked the Mc variation and its error by employing the bootstrap approach. There were several methods to estimate the Mc estimation, such as the goodness-of-fit test (GFT), the entire magnitude range (EMR), and maximum curvature (MAXC) [28,29]. The maximum curvature method was the simplest and most efficient method for calculating the Mc. We analyzed events with magnitudes equal to or larger than the Mc. The MAXC method also gave better Mc estimates that were used to find the spatial frequency magnitude distribution (FMD). MAXC yielded a more vigorous estimate than the least squares regression method [30,31]. To find the Mc, we used a time window of 100 event samples, a 50-event shifting step, and a processing grid cell of 0.1^0^ × 0.1^0^ [31,32]. The *b*-value was considered with the least squares regression analysis, but the presence of even a few massive earthquakes could influence the *b*-value results. Therefore, in this study, the maximum likelihood method (not affected by high magnitude earthquakes) was preferred and used to obtain the desired results [32,33]. To calculate the *b*-value, the maximum likelihood method and a processing grid cell of 0.1^0^ × 0.1^0^ were used in the study area. We required at least 50 events with magnitudes larger than the Mc for determining the *b*-value:

(1) $$b=\;\frac{1}{\mathrm{In}\left(10\right)\left(\bar{M}-M_{c}\right)}$$

where Mc is the magnitude of completeness and $\bar{M}$ denotes the average magnitude of a group of earthquakes with M ≥ Mc.

## 6. Results

**Figure 4:**
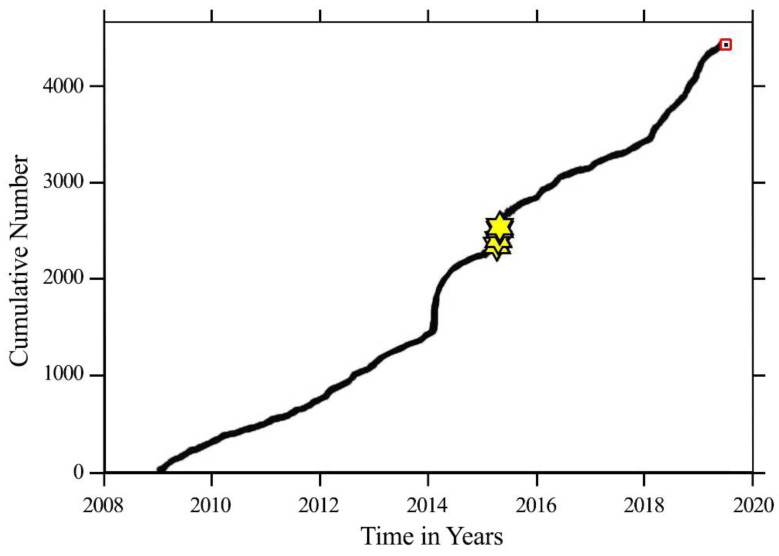
The cumulative number of earthquakes as a function of time. The stars show the earthquakes having a magnitude greater than 7.

We used ZMAP software to calculate the spatial variation of the *b*-value in the study area. First, we tried to calculate the *b*-value over the entire region within the fault blocks. Then, to make the *b*-value calculation results more accurate, we divided the area into three regions based on latitude and longitude. We used magnitude values from 1 to 8.2 to obtain the seismicity map of the entire data catalog for the study area. The map shows the values of earthquakes with magnitudes greater than 7. The hollow spaces in the maps are due to insufficient data. The cumulative number image shows earthquake events as a function of time (Figure 4). We observed that the rate of earthquakes was not constant with time. Earthquakes with the highest magnitude mostly occurred in the year 2015.

**Figure 5:**
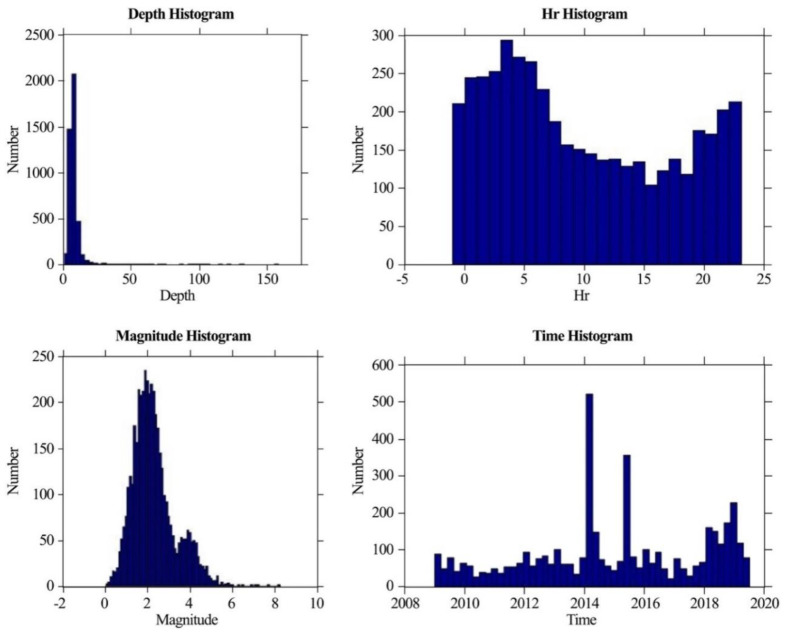
Depth (Km) histogram, hour-of-the-day histogram, magnitude histogram, and time (Year) histogram.

In addition, we calculated different histograms to find different relations with the number of earthquakes, as shown in Figure 5. The depth histogram showed that earthquakes in the region were shallow. Most earthquakes occurred near 20 km, yet only a few events were found at a depth of more than 150 km, which showed that there must be an event at a greater depth. The hour-of-the-day histogram showed that the reporting was uniform throughout the day and night, and we can thus assume that the data were not contaminated by explosions or factors other than fault seismicity. The time histogram shows earthquakes that occurred from 2009 to 2019. The first peak in earthquake activity occurred in 2014–2015, which also contained the Gorkha earthquake (Mw 8.2), while the second peak occurred in 2018–2019. In between these two peaks, there was less fluctuation of events. However, the frequency of several earthquakes was more significant in 2014–2015 as compared with 2018–2019. Finally, we determined the distribution as a function of magnitude by plotting the appropriate histogram. The result showed that the maximum number was near M2, and an organized result of magnitudes ranged from 0.1 to 8.2, as earthquakes with large magnitudes were fewer in number.

**Figure 6:**
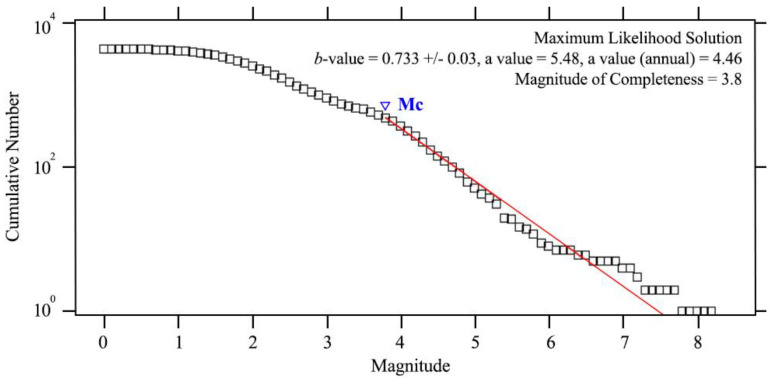
Frequency-magnitude distribution (FMD) of the overall data catalog. The thin line represents the Gutenberg-Richter equation fit.

To calculate the Mc, an alternative presentation in the cumulative form was obtained by first plotting the aggregate number as a function of time and then selecting the Mc and *b*-value estimates. The results estimated the Mc = 3.8 and *b*-value 0.733 +/− 0.03 for the overall catalog (Figure 6). The squares indicate the cumulative number of events for M ≥ m. The line indicates the linear fit to the frequency-magnitude distribution. We chose Mc = 3.8 for the *b*-value computation in the study area for the years 2009 to 2019.

**Figure 7:**
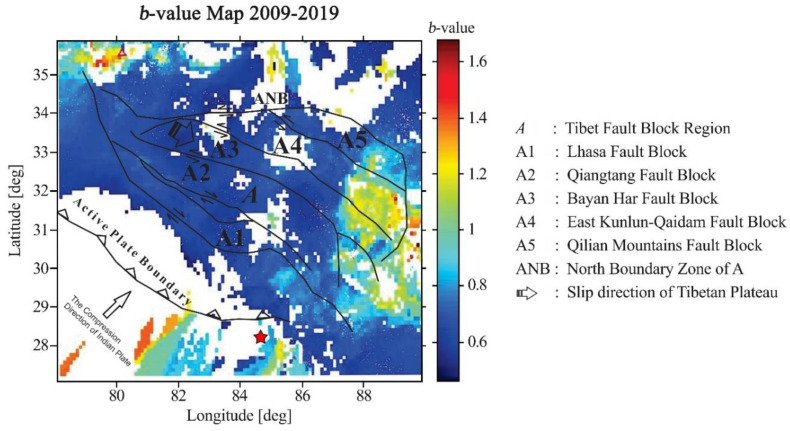
A map of the *b*-value distribution and its relationship with the fault blocks in the study area. The star shows the Gorkha earthquake that occurred in 2015.

Figure 7 shows the *b*-value in the study area, along with fault blocks. The *b*-value is one of the most important statistical parameters, describing the scaling characteristics of the magnitude of the seismic activity. The change in *b*-value was approximately 0.5 to 1.6, depending on the combination of high seismicity and complicated stress patterns. Therefore, the *b*-value in the study area varied. However, FMD results and the *b*-value map showed that the *b*-value in most of the region was approximately 0.6 to 0.9. We found a high *b*-value in the northwest corner and a broad area in the eastern part of the study area. A low *b*-value was present in the center and south of the study area, which likely resulted from the presence of strike-slip faults and underthrusting in that region.

**Figure 8:**
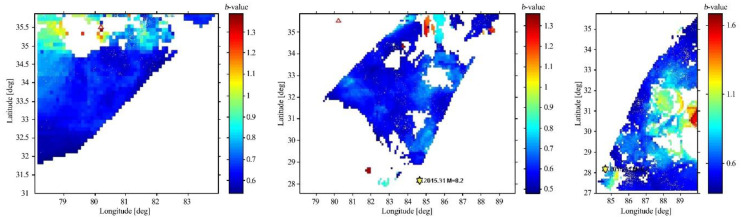
Spatial variation of the *b*-value for Regions 1, 2, and 3. The stars in Regions 2 and 3 show the Gorkha earthquake that occurred in 2015.

The area was also divided by latitude and longitude into three parts, based on the maximum availability of the data in the study area. Regions 1 and 2 were registered as areas with low *b*-values, whereas Region 3 was characterized as having a high *b*-value, as shown in Figure 8. Most of the region showed low *b*-values due to the presence of strike-slip faults in the study area. This relationship proved that Regions 1 and 2 were more prone to seismic activity when compared to region 3.

**Figure 9:**
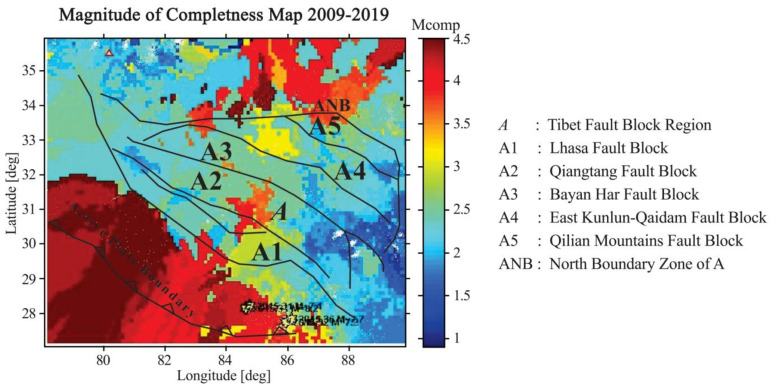
Map of the magnitude of completeness.

The conventional method to analyze the magnitude of completeness (Mc) is to calculate the Mc over the entire period, which is mapped for the region (Figure 9). The map of the Mc value calculated from the earthquake data catalog of 2009–2019 shows Mc values that range from 1 to 4.5. The map shows a high Mc value in the northeastern study area, whereas most of the study area has intermediate Mc values. Most of the area falls under an Mc value of 3.8.

**Figure 10:**
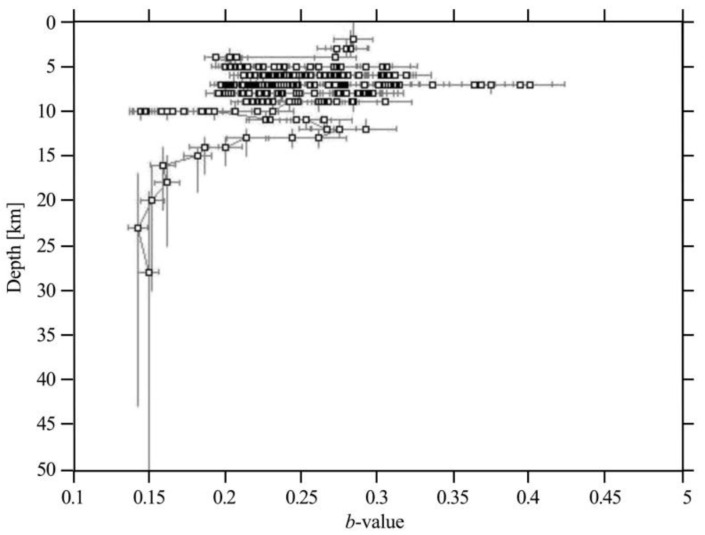
Curve of *b*-value with depth in the study area.

In the *b*-value of the pulled section, we found that the *b*-value in some places did not change systematically with increasing depth. The static pressure of the surrounding rock increased, and the crustal medium was relatively more uniform. Any initial rupture was likely to expand into a large rupture, and the probability of a large earthquake also increased. The discussion describes how the increase of the surrounding rock pressure combined with the increase in the depth has a profound impact on *b*-value distribution. The stratification of the structure in the study area is significant. According to the study, the value of the *b*-value decreased systematically as depth increased [33]. At a depth range greater than 14 km, the *b*-value increased with depth. The earthquake swarm was mainly controlled by a series of strike-slip faults and was less affected by the static pressure of surrounding rocks. The *b*-value was smallest at a depth of 24 km, indicating that the stress was most concentrated near this depth. The most frequent occurrence of large-magnitude earthquakes occurred at depths below 14 km, which also had a low *b*-value (Figure 10). According to previous studies, the *b*-value was shown to decrease with greater depth in the Tibetan Plateau (Xinjiang, Southern Tibet, and Longmenshan area).

## 7. Discussion

The Tibetan Plateau is a young orogenic belt and experiences forces due to the constant movement of the Indian Plate beneath the Eurasian Plate. The tectonic setting of this region is complicated. The *b*-value is usually 1.0, but for shorter time windows, it changes significantly, depending on the tectonic setting of the seismically active region, as was the case from 2009–2019. Apart from compressing the Indian Plate in the south, the Tibetan Plateau and fault blocks within it move southeastward with different rates, due to the difference in the geological framework. The tectonic regime of the Tibetan Plateau consists of sinistral strike-slip faults, dextral strike-slip faults, and normal faults, and it experiences underthrusting in the south. The Tibetan Plateau is the most active continental earthquake region in the world. Due to very few seismic stations and a lack of data, there are many hollow spaces in the study area. Thus, Mc was vital when analyzing earthquake data. The FMD result showed a 3.8 Mc value in the study area. According to our results, the study area had a high magnitude of completeness value in the northeast. The hour-of-the-day histogram showed a constant earthquake rate during the day and at night, which might not be affected due to other sources, such as explosions. The primary purpose of this study was to find the *b*-value in the western part of the Tibetan Plateau and its surrounding areas, which is a useful parameter for predicting the position of future earthquakes, as it relates to the *b*-value changes within the fault blocks region.

The *b*-value is the slope of a fitted line for the power law, and it is effective in revealing the relative occurrences of small and large earthquakes. A high *b*-value indicates a weak region with frequent small events. A low *b*-value indicates an active region overwhelmed by large events. Many factors can cause a perturbation of an average *b*-value (*b*~1.0). Regions with lower *b*-values are subjected to higher applied shear stress after the main shock, whereas regions with higher *b*-values have experienced slip. A high *b*-value from areas with increased geological complexity indicates the importance of multifracture regions. A low *b*-value is related to a low degree of heterogeneity in the fractured medium that experiences high stress, high strain, a high deformation rate, and large faults [22]. A higher *b*-value indicates that an area has a relatively low level of stress accumulation. Small earthquakes are the main future activity. Most faults in the study area are sinistral or dextral strike-slip faults, which lead to the region’s intermediate *b*-values, except in the southern region, where underthrusting produces a low *b*-value. We found a high *b*-value in the northwest corner and a zone in the eastern part of the study area, which showed that there will be small earthquakes in the near future. In this study, *b*-values were between 0.5 and 1.6, and most of the study area showed a low *b*-value of 0.6–0.9. Most earthquakes with magnitudes greater than 7 occurred in an area with a low *b*-value. Low *b*-values are related to generally homogeneous rock properties, which are characterized by large stress and strain, a fractured medium, a high deformation rate, and large faults. According to this value, we can conclude that the area is highly deformed and typified by significantly large stress and strain. The results show that most earthquakes are shallow, and that the *b*-value decreases with depth.

## 8. Conclusions

The theory of *b*-value variation was applied to estimate *b*-values in the Tibetan Plateau and its surrounding areas, which mostly includes strike-slip faults, and assess their *b*-value relationship with fault blocks. The area is highly deformed, consisting of strike-slip faults, normal faults, and thrust faults. The results indicated that most earthquakes occurred during 2014–2015, including the large 2015 Gorkha earthquake. The earthquakes in this area are mostly shallow and of low magnitude; fewer high-magnitude earthquakes occur at greater depths. The variation of *b*-values in this region was calculated, based on the earthquake data catalog from 2009 to 2019. The earthquake with the highest magnitude in this region was 8.2 M. We discovered that the *b*-value in this region ranged from 0.5 to 1.6. The results from the FMD and the *b*-value map indicated that the *b*-value in most of the region was approximately 0.6 to 0.9. Most of the study area is characterized by strike-slip faults, and thus results showed an intermediate value. The map of the magnitude of completeness shows Mc values ranging from 1 to 4.5. Further, FMD gave an overall Mc value of 3.8. We assumed that areas located in the south were more prone to earthquakes because they were located near the collision boundary of the two plates. This area showed a low *b*-value when compared to other regions. Areas of the study region showing high *b*-values will be prone to small earthquakes in the near future. Low *b*-values revealed the degree of variation in rock properties, including large stress and strain, a fractured medium, a high deformation rate, and large faults. Where the stress level is high in the region under investigation, low or smaller *b*-values were observed, which could cause a massive high-magnitude earthquake. Recent studies [34] have used natural time analysis [35] and showed that a decrease in *b*-value reflected an increase in the order parameter fluctuations of seismicity. This new analysis identified useful precursory phenomena before major earthquakes [36] and other complex systems [37]. Therefore, it is necessary to have more seismic station coverage in this area so that more data can be acquired and interpreted. Field data should be obtained to study the major faults in the area, which is difficult due to the rough terrain and harsh environment.

## Figures and Tables

**Table 1:** The number (N) of earthquakes in a specific magnitude (M) range.

**M**	**0.1**	**0.2**	**0.3**	**0.4**	**0.5**	**0.6**	**0.7**	**0.8**	**0.9**	**1.0**	**1.1**	**1.2**
**N**	2	4	10	16	15	20	37	41	64	76	107	119
**M**	**1.3**	**1.4**	**1.5**	**1.6**	**1.7**	**1.8**	**1.9**	**2.0**	**2.1**	**2.2**	**2.3**	**2.4**
**N**	111	174	156	214	208	212	235	223	209	220	212	187
**M**	**2.5**	**2.6**	**2.7**	**2.8**	**2.9**	**3.0**	**3.1**	**3.2**	**3.3**	**3.4**	**3.5**	**3.6**
**N**	172	145	129	98	91	76	66	55	41	35	47	53
**M**	**3.7**	**3.8**	**3.9**	**4.0**	**4.1**	**4.2**	**4.3**	**4.4**	**4.5**	**4.6**	**4.7**	**4.8**
**N**	51	51	61	58	49	49	47	32	23	21	18	21
**M**	**4.9**	**5.0**	**5.1**	**5.2**	**5.3**	**5.4**	**5.5**	**5.6**	**5.7**	**5.8**	**5.9**	**6.0**
**N**	11	8	6	6	11	1	4	1	2	3	1	1
**M**	**6.3**	**6.5**	**6.9**	**7.1**	**7.2**	**7.7**	**8.2**					
**N**	1	1	1	1	1	1	1					
